# Neuroanatomical Analysis of the Carpus and the Base of the Thumb: Advancing Denervation-Based Interventions

**DOI:** 10.7759/cureus.89142

**Published:** 2025-07-31

**Authors:** Benjamin F Watzig, Lloyd Champagne, Molly Sekar, Clayton Hui, James Cardinal, Stephanie Nulty, Tony Huynh, Matthew Eisenberg, Joshua W Hustedt

**Affiliations:** 1 Orthopedic Surgery, University of Arizona College of Medicine, Phoenix, USA; 2 Plastic Surgery, Arizona Center for Hand to Shoulder Surgery, Phoenix, USA

**Keywords:** denervation, innervation, neuroanatomy, osteoarthritis, pain

## Abstract

Background: Joint denervation procedures continue to demonstrate promise in the management of chronic pain and functional improvement in joint pathology of the hand and wrist. As our understanding of these techniques evolves, a detailed comprehension of neuroanatomy, including the precise relationships and contributions of sensory innervation to targeted joints, is critical for optimizing outcomes.

Methods: Freshly thawed frozen upper extremity cadaveric specimens were analyzed under the direction of two fellowship-trained hand surgeons. Each extremity was dissected from the mid-humerus to the fingertip, following all named nerve branches distally to their specific innervation targets, including the first carpometacarpal (CMC) joint, radiocarpal joint (RCJ), triangular fibrocartilage complex (TFCC), and distal radioulnar joint (DRUJ). Under loupe magnification, each target was evaluated based on the following criteria: direct innervation with visualized nervous interdigitation, close proximity (<2 mm) without visualized innervation, and proximity >2 mm or not visualized.

Results: Ten cadaveric specimens were dissected. The first CMC joint was innervated by the recurrent branch of the median nerve (RBM) in 10 (100%) cases. Additionally, the lateral antebrachial cutaneous nerve (LABCN), superficial branch of the radial nerve (SBRN), and palmar cutaneous branch of the ulnar nerve (PCBUN) directly innervated the CMC joint in eight (80%) cases and were in close proximity in the remaining two (20%). The RCJ was innervated either directly or in close proximity in at least eight (80%) specimens by the anterior interosseous nerve (AIN), posterior interosseous nerve (PIN), LABCN, PCBUN, and SBRN. In all 10 (100%) specimens, the DRUJ was innervated either directly or in close proximity by the AIN, the PIN, the dorsal cutaneous branch of the ulnar nerve (DCBUN), or the PCBUN. The TFCC was innervated either directly or in close proximity in all 10 (100%) by PIN, AIN, and DCBUN. The TFCC was directly innervated by PCBUN in only five (50%) specimens, while the medial antebrachial cutaneous nerve was observed in close proximity in two (20%).

Conclusion: Despite slight variability between specimens, a general pattern of innervation for the first CMC, RCJ, TFCC, and DRUJ was observed. Our results provide recommendations for specific nerve targets in the wrist to treat chronic pain and arthritis. As our understanding of these neuroanatomic structures and patterns continues to expand, so too will our ability to tailor denervation procedures to patient-specific pathologies.

## Introduction

Targeted denervation has emerged as an innovative and evolving treatment modality for managing chronic pain and arthritis. By selectively ablating sensory nerves, this approach disrupts afferent pain signaling pathways and allows for intervention that can be motion or joint-sparing [1]. Research continues to emerge supporting its efficacy in a variety of conditions, specifically the first carpometacarpal (CMC), radiocarpal, and knee, where it has been shown to deliver significant pain relief and measurable improvements in patient-reported functional outcomes [2-4].

The success of targeted denervation hinges on a detailed understanding of sensory innervation, particularly in the wrist and hand. The first CMC joint, the radiocarpal joint (RCJ), triangular fibrocartilage complex (TFCC), and distal radioulnar joint (DRUJ) are critical structures implicated in chronic pain syndromes from a variety of pathologies. As literature continues to arise regarding surgical denervation techniques, we require refinement of the anatomic spatial relationships and the variation amongst the population. Prior work has noted a complex interplay of afferent signaling from the anterior and posterior interosseous nerves (AIN, PIN), the superficial branch of the radial nerve (SBRN), the lateral antebrachial cutaneous nerve (LABCN), and branches of the ulnar nerve (palmar cutaneous branch of the ulnar nerve (PCBUN), dorsal cutaneous branch of the ulnar nerve (DCBUN)) [5,6].

The goal of this study is to comprehensively characterize the sensory innervation of the first CMC, RCJ, TFCC, and DRUJ through meticulous dissection and nerve tracing. Our objective was to evaluate the terminal articular targets of identified afferent nerves and their proximity to these structures in cadaveric specimens using gross anatomical analysis. By delineating the pathways of proximal nerve branches to their distal articular destinations, we aim to refine and develop patient-specific recommendations for denervation based on anatomical location and commonly observed patterns of pathology.

## Materials and methods

The study was conducted at the University of Arizona College of Medicine - Phoenix, at Banner University Medical Center. Freshly thawed frozen upper extremity cadaveric specimens were analyzed under the direction of two fellowship-trained hand surgeons. Each extremity was dissected from the mid-humerus to the fingertip, following all named nerve branches distally to their specific innervation targets, including the first CMC joint, RCJ, TFCC, and DRUJ (Table 1). Under loupe magnification, each target was evaluated based on the following criteria: direct innervation with visualized nervous interdigitation, close proximity (<2 mm) without visualized innervation, and proximity >2 mm or not visualized. Each dissection was independently reviewed by the two surgeons, with a third serving as a tiebreaker in cases of disagreement.

**Table 1:** Nerve and innervation targets

Acronym	Definition
Nerve structure	
AIN	Anterior interosseous nerve
DCBUN	Dorsal cutaneous branch of the ulnar nerve
LABCN	Lateral antebrachial cutaneous nerve
MACN	Median cutaneous nerve of the arm
PCBUN	Palmar cutaneous branch of the ulnar nerve
PIN	Posterior interosseous nerve
RBM	Recurrent branch of the median nerve
RBU	Recurrent branch of the ulnar nerve
SBRN	Superficial branch of the radial nerve
Innervation target	
CMC	Carpometacarpal joint
DRUJ	Distal radioulnar joint
RCJ	Radiocarpal joint
TFCC	Triangular fibrocartilage complex

A total of 10 cadaveric upper extremities were included in this study, six right and four left. Dissections were performed in a controlled environment using standard microsurgical techniques to ensure accurate nerve identification. Documentation of each nerve branch and its relationship to the targeted joints was conducted through high-resolution photography and detailed anatomic drawings. Statistical analysis was performed to determine the prevalence of direct versus indirect innervation for each joint. 

## Results

**Figure 1:**
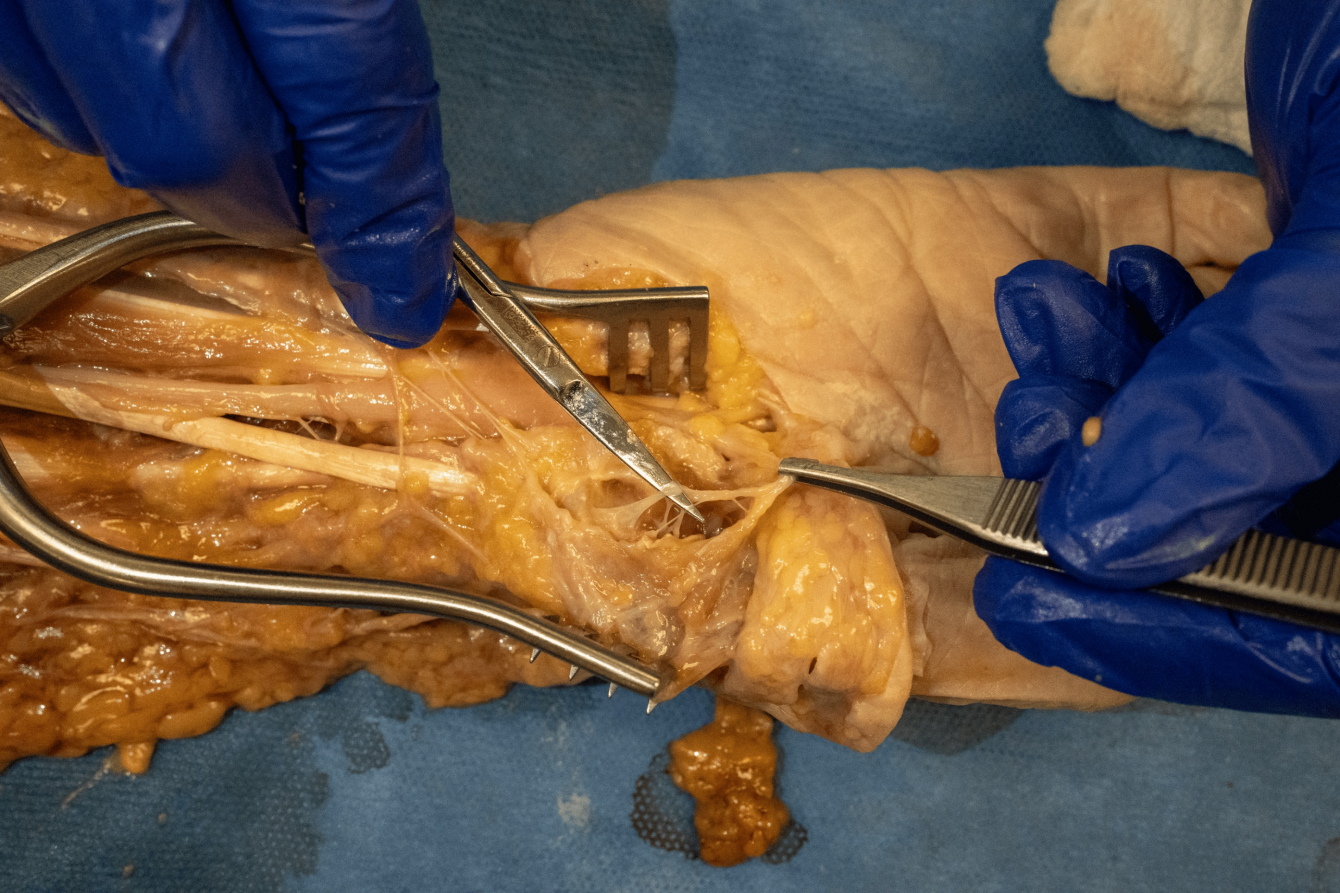
Recurrent median nerve and innervation target Volar view of the wrist on the radial aspect with direct innervation of the carpometacarpal by the recurrent median nerve.

The first CMC joint was innervated by the recurrent branch of the median nerve (RBM) in 10 (100%) of the cadavers (Table 2) (Figure 1). Additionally, the LABCN, SBRN, and PCBUN directly innervated the CMC joint in eight (80%) specimens and were in close proximity in the remaining two (20%).

**Table 2:** Anatomic structures with associated afferent nerve targets TFCC, DRUJ, RCJ, and CMC structures with associated innervations N(%). TFCC: triangular fibrocartilage complex; PIN: posterior interosseous nerve; AIN: anterior interosseous nerve; DCBUN: dorsal cutaneous branch of ulnar nerve; LABCN: lateral antebrachial cutaneous nerve; MACN: median cutaneous nerve of arm; PCBUN: palmar cutaneous branch of ulnar nerve; PIN: posterior interosseous nerve; RBM: recurrent branch of median nerve; RBU: recurrent branch of ulnar nerve; SBRN: superficial branch of radial nerve; CMC: carpometacarpal joint; DRUJ: distal radioulnar joint; RCJ: radiocarpal joint

Structure	Direct innervation N(%)	Close proximity N(%)	Not visualized N(%)
TFCC			
PIN	9(90)	1(10)	0(0)
DCBUN	10(100)	0(0)	0(0)
PCBUN	5(50)	0(0)	5(50)
AIN	9(90)	1(10)	0(0)
MACN	0(0)	2(20)	8(80)
DRUJ			
AIN	10(100)	0(0)	0(0)
PIN	8(80)	2(20)	0(0)
DCBUN	8(80)	2(20)	0(0)
PCBUN	10(100)	0(0)	0(0)
RCJ			
AIN	9(90)	0(0)	1(10)
PIN	10(100)	0(0)	0(0)
LABCN	4(40)	6(60)	0(0)
PCBMN	7(70)	1(10)	2(20)
SBRN	6(60)	2(20)	2(20)
CMC			
LABCN	8(80)	2(20)	0(0)
SBRN	8(80)	2(20)	0(0)
PCBMN	8(80)	2(20)	0(0)
RBM	10(100)	0(0)	0(0)
RBU	0(0)	0(0)	10(10)

**Figure 2:**
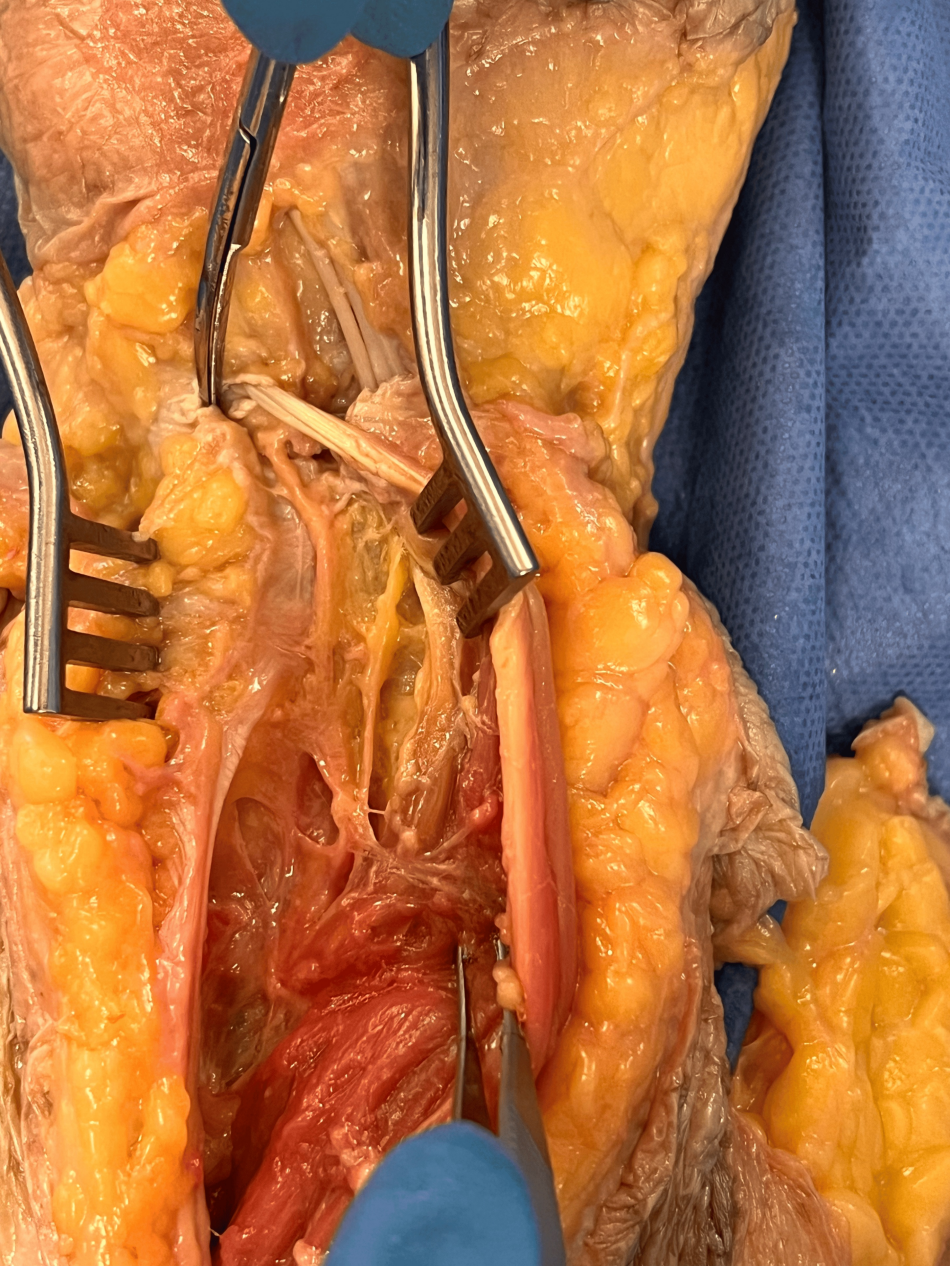
PIN innervation targets Dorsal view of the wrist with PIN branching to DRUJ and TFCC on the left side of the image and RCJ on the right. PIN: posterior interosseous nerve; DRUJ: distal radioulnar joint; TFCC: triangular fibrocartilage complex; RCJ: radiocarpal joint

**Figure 3:**
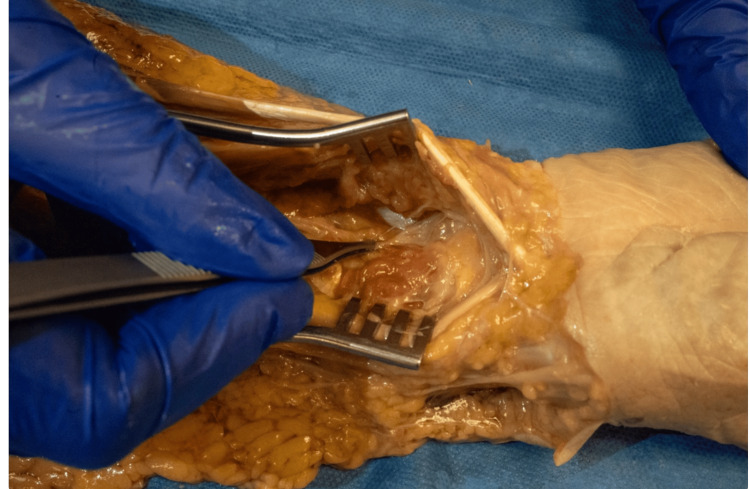
AIN and innervation target Volar view of the wrist with AIN branching to the DRUJ. AIN: anterior interosseous nerve; DRUJ: distal radioulnar joint

The RCJ was innervated either directly or in close proximity in eight (80%) specimens by the AIN, PIN (Figure 2), LABCN, PCBUN, and SBRN. The DRUJ demonstrated in 10 (100%) cases had direct or close-proximity innervation by AIN (Figure 3), PIN, DCBUN, or PCBUN. The TFCC was innervated either directly or in close proximity in 10 (100%) cases by PIN, AIN, and DCBUN. The TFCC was directly innervated by PCBUN in only five (50%) of the cases, while the medial antebrachial cutaneous nerve was observed in close proximity in two (20%) of the specimens.

Following the dissection of the 10 cadaveric specimens, there was complete agreement between the two primary reviewers in nine upper extremities. For one specimen, a third-hand surgeon served as a tiebreaker to resolve a disagreement regarding whether the PIN demonstrated direct innervation of the TFCC or was merely in close proximity. 

## Discussion

Our cadaveric study contributes to the growing body of literature delineating the complex sensory innervation of the wrist and thumb CMC joint. We documented the frequency with which each identified afferent nerve was observed in relation to its target, whether in direct contact, in close proximity without direct innervation, or not visualized at all. This detailed mapping provides critical information to guide future protocols for selective nerve ablation or transfer in the management of regional pain. As we continue to understand these neural targets, we can refine patient-specific denervation procedures to treat pain and improve function with preservation of native target structures. 

These data corroborate with previous studies identifying the AIN and PIN as significant contributors to the articular innervation of the RCJ, DRUJ, and TFCC, while also demonstrating consistent contributions from cutaneous branches of the radial and ulnar nerves to the CMC joint [5,7].

The AIN and PIN have emerged as key surgical targets for denervation of the wrist joint. Previous studies have shown that selective neurectomy of these branches can result in significant pain relief and functional improvement in patients with radiocarpal arthritis [8,9]. Our dissection findings support these clinical observations and offer an anatomical rationale for targeting these nerves. In particular, the AIN was found to provide articular branches not only to the RCJ but also to the DRUJ and TFCC structures frequently implicated in ulnar-sided wrist pain.

Additionally, our study reinforces the notion that the four main denervation targets were innervating the first CMC joint. In all specimens, the LABCN, SBRN, PCMN, and RBM nerves were either directly innervated or were in close proximity. While some of these nerves have traditionally been considered cutaneous, recent anatomical and clinical investigations have identified their articular branches, suggesting a broader role in joint sensory innervation than previously recognized [2,7,10].

Given the anatomic nature of the study, it is not without limitations. As with all cadaveric investigations, anatomical variability and specimen preservation can influence results. Although the number of cadavers dissected is comparable to other reported studies in the literature, the relatively small sample size may limit the statistical power and generalizability of our findings. Additionally, demographic information such as age and sex of the specimens was not available, which could affect the morphological variation seen. Furthermore, nerve proximity assessments, particularly for distances less than 2 mm, were based on visual estimation, which may introduce observer variability and limit the precision of our results. Finally, the study relies solely on gross anatomical dissection without histological confirmation, which limits validation of the presence of small nerve fibers at terminal insertion sites. 

Our study provides detailed anatomical evidence of the sensory nerve supply to the first CMC joint, RCJ, DRUJ, and TFCC. These findings establish a structural foundation for targeted denervation procedures and aim to support surgeons in incorporating nerve-specific approaches in the treatment of wrist and thumb base pain. However, as the data are anatomical and descriptive, the clinical relevance, particularly regarding improved surgical outcomes, remains speculative. Further clinical studies are essential to correlate these anatomical patterns with patient outcomes. Such investigations will be critical for validating the practical utility of these findings in surgical practice, refining patient selection criteria, and ultimately optimizing the safety and efficacy of denervation procedures.

## Conclusions

Despite slight variability between specimens, a general pattern of innervation for the first CMC, RCJ, DRUJ, and TFCC was observed. Our results provide recommendations for specific nerve targets in the wrist to treat chronic pain and arthritis. As our understanding of these neuroanatomic structures and patterns continues to expand, so too will our ability to tailor denervation procedures to patient-specific treatments.
